# Temporal Predictors of Health-Related Quality of Life in Elderly People with Diabetes: Results of a German Cohort Study

**DOI:** 10.1371/journal.pone.0031088

**Published:** 2012-01-24

**Authors:** Imad Maatouk, Beate Wild, Daniela Wesche, Wolfgang Herzog, Elke Raum, Heiko Müller, Dietrich Rothenbacher, Christa Stegmaier, Dieter Schellberg, Hermann Brenner

**Affiliations:** 1 Department of General Internal Medicine and Psychosomatics, Medical University Hospital Heidelberg, Heidelberg, Germany; 2 Division of Clinical Epidemiology and Aging Research, German Cancer Research Center, Heidelberg, Germany; 3 Institute for Epidemiology and Medical Biometry, University of Ulm, Ulm, Germany; 4 Saarland Cancer Registry, Saarland Ministry of Public Health, Saarbrücken, Germany; Fundación para la Prevención y el Control de las Enfermedades Crónicas No Transmisibles en América Latina (FunPRECAL), Argentina

## Abstract

**Background:**

The aim of the study was to determine predictors that influence health-related quality of life (HRQOL) in a large cohort of elderly diabetes patients from primary care over a follow-up period of five years.

**Methods and Results:**

At the baseline measurement of the ESTHER cohort study (2000–2002), 1375 out of 9953 participants suffered from diabetes (13.8%). 1057 of these diabetes patients responded to the second-follow up (2005–2007). HRQOL at baseline and follow-up was measured using the SF-12; mental component scores (MCS) and physical component scores (PCS) were calculated; multiple linear regression models were used to determine predictors of HRQOL at follow-up. As possible predictors for HRQOL, the following baseline variables were examined: treatment with insulin, glycated hemoglobin (HbA1c), number of diabetes related complications, number of comorbid diseases, Body-Mass-Index (BMI), depression and HRQOL. Regression analyses were adjusted for sociodemographic variables and smoking status. 1034 patients (97.8%) responded to the SF-12 both at baseline and after five years and were therefore included in the study. Regression analyses indicated that significant predictors of decreased MCS were a lower HRQOL, a higher number of diabetes related complications and a reported history of depression at baseline. Complications, BMI, smoking and HRQOL at baseline significantly predicted PCS at the five year follow-up.

**Conclusions:**

Our findings expand evidence from previous cross-sectional data indicating that in elderly diabetes patients, depression, diabetes related complications, smoking and BMI are temporally predictive for HRQOL.

## Introduction

In the European Union (EU)-25 States, the proportion of people aged 65 and older is expected to rise substantially, from 16.4% in 2004 to 29.9% in 2050 [1]. Similar demographic changes are seen in many other developed countries and will also be accompanied by an increasing prevalence of chronic diseases such as diabetes [2] leading to a deterioration of quality of life [3].

Health-related quality of life (HRQOL) refers to those aspects of quality of life that relate to a person's perception of health. HRQOL is influenced not only by a person's health status, but by their ability to cope with the burden of disease. The concept of HRQOL includes various domains that refer to the physical, psychological, and social domains of health. Important components of HRQOL, as conceptualized, are: physical functioning, mental health, bodily pain, general health, vitality, and social functioning [4]–[6].

The importance of HRQOL as a relevant outcome variable in diabetes has increasingly been recognized; and reduced HRQOL has been hypothesized as an independent marker of mortality [7]. Most of the previous studies were based on cross-sectional samples; in addition, only a very few studies focused on elderly adults. The results of these cross-sectional studies have been rather inconsistent. However, the occurrence of diabetes-related complications seems to be clearly associated with a decline in HRQOL. Conversely, the cross-sectional relationship between HRQOL and glycemic control or treatment regimen remains unclear [8]–[10].

What remains unknown is: Which temporally preceding factors influence HRQOL in elderly adults with diabetes? To date, only a few prospective studies regarding HRQOL in diabetes patients have been reported. In a clinical sample of 3642 adults with a mean age of 53 years, Stratton et al. [11] reported that higher glycated hemoglobin (HbA1c) levels (a marker for average blood glucose levels over the previous two months) longitudinally predict higher complication rates that, in turn, could affect HRQOL. However, Weinberger et al. [12] for instance, found no significant association between glycemic control at baseline and HRQOL. Results of a large population-based study showed that age, female sex, lower educational level, obesity, and no physical activity were temporally predictive of lower self-rated health [13]. However, in this study the multidimensional construct of quality of life has not been adequately assessed by a validated questionnaire such as the Short-Form General Health Survey (SF-36, SF-12).

In order to gain more knowledge which determinants longitudinally predict HRQOL in elderly adults with diabetes, population-based studies are needed to improve the quality of care of that expanding group. The aim of this study was to determine predictors that influence HRQOL in elderly patients with diabetes over a follow-up period of five years. Risk factors hypothesized to be associated with reduced HRQOL were investigated in a prospective cohort study. All analyses were adjusted for sociodemographic variables and smoking status.

## Methods

### Design and study population

In the present study, we analysed data from the baseline and second follow-up measurement of the population-based prospective ESTHER cohort study - a population-based cohort study of older adults conducted in Saarland, a state located in South West Germany. Between July 2000 and December 2002, in the federal state of Saarland, 9953 participants aged 50 to 74 years were recruited by their general practitioners (GPs) in the course of a health check-up that focused on early detection of chronic diseases. In Germany this health check-up is offered biennially to older adults. Baseline data shows that the ESTHER study sample is representative with respect to both demographic variables and chronic diseases of the general German population [14]. The study was approved by the Ethics Board of the University of Heidelberg and by the Ethics Board of the medical association of the State of Saarland. Written informed consent was obtained from all participants.

At baseline, participants completed a comprehensive self-administered questionnaire including the SF-12 to assess HRQOL; blood and urine samples were taken. The medical information was reported by the GPs. Between July 2005 and December 2007, 8270 elderly people aged 53 to 80 years participated in a second follow up ( = 90.0% response rate of those still alive). In the second follow-up, participants again completed a self-administered questionnaire that included the SF-12.

### Measurements

In the predictor analysis of HRQOL in diabetes patients, the following baseline variables were included: HbA1c, treatment with insulin, number of complications, number of comorbid diseases, BMI, self-reported history of current or previous depression, HRQOL at baseline, gender, age, nationality, marital status, educational level, and smoking.

HRQOL at baseline and follow-up was measured with the SF-12- Questionnaire. The SF-12 is a widely used generic questionnaire that does not focus on specific disease groups. Several domains of HRQOL – such as physical, social and role functioning – are assessed. Items are weighted and totalled to provide both physical (PCS) and mental component scores (MCS) ranging from 0 to 100. A higher score in the respective summary scales indicates a higher quality of life. The questionnaire shows good psychometric criteria [5].

Diabetes was defined as follows: diabetes mellitus documented by the GP on the standardized health check-up form, or the use of anti-diabetic medication.

HbA1c is an established biochemical marker of diabetes control over the preceding two months. HbA1c analyses at baseline were done using high performance liquid chromatography in a central laboratory. Urinary albumin concentration was measured by immunonephelometry assay in a spot urine sample. The following diabetes-related complications were counted: cardiac complications (self-reported angina pectoris or physician-recorded coronary heart disease or self-reported myocardial infarction), self-reported history of stroke, self-reported circulatory disorder of the legs and nephropathy; the variable thus ranged from 0 (if no complications were recorded/reported) to 4 (if all four complications were recorded/reported). Nephropathy as a complication of diabetes was defined by albuminuria (urinary albumin≥20 mg/l). The following comorbid diseases (other than diabetes-related complications) were counted separately: physician-recorded hypertension, physician-recorded dyslipidemia, self- reported history of asthma, and self-reported history of cancer (composite variable ranging from 0 to 4). Self-reported depression was classified into “no” (no depression has ever been diagnosed) and “yes” (current or previous depression episode has been diagnosed). The participants' self-reported weight (in kilograms) and height (in metres) were used to calculate BMI (body mass index = weight in kilograms divided by height in metres squared). BMI and age were treated as continuous variables. Marital status was classified into “married” (referent), “divorced/widowed” and “unmarried”. Duration of school education was categorized into the categories ≤8, 9–10 (referent), 11–12, and >12 years. Cigarette smoking was classified into “never” (referent), “former” or “current” (variable ranging from 0 to 2).

### Statistical analyses

In general, the mental and physical component summary scores (MCS and PCS) of the SF-12 are computed by using a linear weighting of the 12 items. However, if one or more SF-12 items are missing, a sophisticated algorithm has to be applied to replace missing values. In our study, 64.0% of the participants completed all of the SF-12 items both at baseline and after five years; 36.0% of the participants had one or more missing items in the SF-12 questionnaires. To deal with missing items, the robust modified regression estimation (MRE) methods were used [15], [16]. In 2004, Spiro and colleagues published a detailed technical report regarding the imputing of PCS and MCS for the Veterans SF-12 Health Survey (VR-12). The authors presented a sophisticated algorithm (including SAS macros) for estimating PCS and MCS in the context of missing values. However, Spiro et al. used the VR-12 form to measure HRQOL that is slightly different from the SF-12 version regarding the answer- format of four specific items. All 12 items of SF-12 and VR-12 are identically formulated.

However, there are four items - regarding “role physical” (RP) and “role emotional” (RE) - that differ in their response format between SF-12 and VR-12.

The two items regarding RP are:

- “During the past 4 weeks, have you had any of the following problems with your work or other regular daily activities as a result of your physical health?”
  - “Accomplished less than you would like”
  - “Were limited in the kind of work or other activities”.

The two items regarding RE are:

- “During the past 4 weeks, have you had any of the following problems with your work or other regular daily activities as a result of any emotional problems (such as feeling depressed or anxious)?”
  - “Accomplished less than you would like”
  - “Didn't do work or other activities as carefully as usual”

For each of these four items the SF-12-response format is dichotomized: yes/no. In contrast the VR-12-response format comprises a 5-point Likert scale: none of the time, a little of the time, some of the time, most of the time, all of the time.

The SF-12 does not tolerate any missing item whereas incomplete responses in VR-12 can be dealt with with the MRE. In order to apply the MRE-method, we converted the responses of the four SF-12-items diverging in response format to the VR-12 response format. The SF12-response “yes” was converted to the VR-12-response format “most of the time” while the SF-12-response “no” was converted to the VR-12 response “a little of the time”. As a validation check for the transformation algorithm the correlation between SF-12 scale scores and VR-12 scale scores was computed and showed a high correlation (r>0.99). After transforming the SF-12 scoring format into the VR-12 format, it was possible to use the already published imputation methods for the missing values. Depending on the missing-pattern at hand, the algorithm selects the corresponding imputation model from the database. In order to allow the imputing process to take place with only a minimum level of accuracy, the minimum R-square value required was kept at the default-value of 0.6.

Regression analyses were conducted separately for the dependent variables MCS and PCS. For the linear regression analysis to predict MCS and PCS respectively after five years a four-step approach was applied. Firstly, a reference model was defined comprised of a set of control variables: gender, age, nationality, marital status, educational level, and smoking. Secondly, from the pool of the ESTHER data, the following variables of interest for a prognostic model were selected (guided by theory and literature [9], [17]): HbA1c, count of diabetes-related complications (self-reported angina pectoris, physician-recorded coronary heart disease, self-reported myocardial infarction, self-reported history of stroke, self-reported circulatory disorder of the legs, nephropathy), count of comorbid diseases (physician-recorded hypertension, physician-recorded dyslipidemia, self- reported history of asthma, self-reported history of cancer), self-reported depression, BMI, and treatment with insulin. Thirdly, univariate analyses were conducted with the selected variables. The associations between PCS (or MCS) and the variables of interest were measured by calculating the Pearson Correlation coefficient. The t-test was used to calculate p-values. Variables yielding p-values<.20 in the correlation analysis were retained as candidate variables for the multiple regression analysis. We used the level of 0.20 as a screening criterion for selection because a more traditional level (such as 0.05) could fail to identify variables known to be important [18], [19]. Finally, candidate variables were entered into a multivariable regression model that already contained the control variables of the reference-model. All multivariate analyses were adjusted for baseline PCS and baseline MCS. Thus, in the final model, the effects of the prognostic variables of interest were adjusted for the effect of the reference-model variables. For all variables, standardized β-coefficients were calculated. All statistical analyses were performed using SAS (version 9.2) for Windows.

## Results

At the baseline measurement of the ESTHER study, 1375 out of 9953 participants suffered from diabetes (13.8%; 95% CI = [13.1; 14.5]). 1057 of these diabetes patients responded to the second-follow up at five years later. At baseline we observed that 414 of 1375 participants had incomplete SF-12 responses. The majority of missing-patterns comprised the case of only one (n = 147) or two (n = 118) missing items. At follow-up after five years we observed that 337 of 1057 cases had incomplete SF-12 responses. The majority of missing-patterns were also one (n = 202) or two (n = 68) missing items.

For 97.8% of the responders of the diabetes patients MCS and PCS were calculated both at baseline and after five years. These 1034 patients were included in the study. Further demographic characteristics of the study participants are shown in **Table 1** **.** Most participants were retired (76.1%); 43.1% had an HbA1c higher than 7%, mean HbA1c was 7.0% (STD = 1.3). 17.2% were under treatment with insulin, all other participants received oral treatment.

**Table 1:** Characteristics of study sample at baseline (n = 1034).

Category	Variable	n	%
Gender	Male	549	53.1
	Female	485	46.9
Nationality	German (yes)	1011	97.8
Age (years)	50–59	250	24.2
	60–64	288	27.9
	65–69	298	28.8
	70–75	198	19.1
Marital Status	Unmarried	41	4.0
	Married	763	73.8
	Divorced, widowed	206	19.9
Duration of school education (years)	0 – 8	41	4.0
	9–10	855	82.7
	11–12	61	5.9
	>12	38	3.7
Working status	Currently working (yes)	187	18.1
Smoking status	Currently smoking	140	13.5
	Former smoker	397	38.4
	Never smoked	467	45.2
Glycemic control (HbA1c %)	<6,1%	247	23.9
	≥6,1%; <7%	333	32.2
	≥7%	446	43.1
Treatment	Insulin (yes)	178	17.2
Comorbidity	Physician diagnosed hypertension	786	76.0
	Physician diagnosed dyslipidemia	544	52.6
	Self-reported cancer	77	7.4
	Self-reported asthma	61	5.9
	Self-reported depression	131	12.7
Diabetes-related Complications	Cardiac complications*	284	27.5
	Stroke	63	6.1
	Circulatory disorder of the legs	248	24
	Albuminuria (≥20mg/l)	237	22.9

Baseline variables were choosen as predictor variables for multiple linear regression analysis (Table 3 and Table 4).

*(includes self reported angina pectoris or myocardial infarction or physician recorded coronary heart disease).

The mean MCS of the 1034 included diabetes patients at second follow-up was 47.9 (STD = 10.2), with MCS values ranging from 13.6 to 65.7. Mean PCS at follow-up was 38.8 (STD = 10.3; range 13.0 to 57.3). In the univariate correlation analyses, all of the selected baseline variables correlated with both MCS and PCS after five years with p<0.20 and were included in the multiple linear regression models. Baseline and 5-year-follow-up values of MCS and PCS are shown in **Table 2** **.**

**Table 2:** Baseline and 5-year-follow-up values of MCS and PCS.

	Baseline	5-year-follow-up
Mental component score (MCS) (Mean±SD)	45.8±10.3	47.9±10.2
Physical component score (PCS) (Mean±SD)	38.9±9.8	38.8±10.3

Component scores of 5-year follow up were chosen as outcome variables of multiple linear regression analyses (Table 3 and Table 4).

As mentioned above, our dependent (outcome) variables were MCS after five years and PCS after five years respectively. The independent (predictor) variables are listed in Table 3 (for MCS) and in Table 4 (for PCS) respectively. The unstandardized regression coefficients are labeled “B”. The usual interpretation of a regression coefficient is the average change in the outcome variable when the corresponding predictor variable is changed by one unit. When the independent variables are measured in different units of measurement, the unstandardized coefficients do not allow direct comparison between predictor variables. To deal with this problem, standardization of regression coefficients is done by multiplying “B” by the standard deviation of the predictor variable and dividing it by the standard deviation of the outcome variable. The resulting standardized regression coefficients are labelled “β”. Standardized coefficients are interpreted as the standard deviation change in the dependent variable when the independent variable is changed by one standard deviation. The standardized coefficients permit direct comparison of the influence between predictor variables. The comparison is between changes in standard deviations instead of changes in the different units of the variables.

**Table 3:** Results of the multiple linear regression analysis with several independent baseline variables predicting MCS at five years later.

Variable	B*	SE†	β‡	p-value
Age **(years)**	0.033	0.052	0.020	0.531
Sex	0.541	0.694	0.026	0.436
Nationality	1.568	2.213	0.021	0.479
Unmarried	−0.456	1.508	−0.009	0.762
Divorced/Widowed	−0.750	0.812	−0.029	0.356
Duration of school education (years)				
0–8 years	−1.339	1.510	−0.026	0.376
11–12 years	1.911	1.236	0.046	0.123
>12 years	0.402	1.560	0.008	0.797
Smoking status	−0.072	0.457	−0.005	0.876
Insulin (yes)	0.483	0.832	0.018	0.562
**Diabetes-related complications**	**−0.748**	**0.372**	**−0.064**	**0.045**
Comorbidities	−0.390	0.405	−0.029	0.336
BMI (kg/m^2^)	−0.018	0.073	−0.010	0.810
**Self-reported depression**	**−2.756**	**0.987**	**−0.088**	**0.005**
**MCS at baseline**	**0.380**	**0.033**	**0.374**	**<0.001**
**PCS at baseline**	**0.112**	**0.034**	**0.107**	**0.001**
HbA1c (%)	−0.378	0.233	−0.049	0.106

Coding of categorical variables is mentioned in the footnote.

*unstandardized regression coefficient; † standard error; ‡ standardized regression coefficient; statistically significant associations are printed in bold

Sex: male and female (referent); Nationality: German nationality (referent), other nationalities. Marital status: “married” (referent), “divorced/widowed” and “unmarried”. Duration of school education: ≤8, 9–10 (referent), 11–12, and >12 years. Smoking status: variable ranging from 0 to 2, classified into “never”, “former” or “current”. Treatment with insulin: “yes” and “no” (referent). Diabetes-related complications: variable ranging from 0 (no complications) to 4 (four complications). Comorbid diseases: composite variable ranging from 0 to 4. Depression: “no” (referent) and “yes” (depression episode has been diagnosed).

**Table 4:** Results of the multiple linear regression analysis with several independent baseline variables predicting PCS at five years later.

Variable	B*	SE†	β‡	p-value
**Age**	**−0.158**	**0.046**	**−0.096**	**<0.001**
Sex	0.863	0.611	0.042	0.158
Nationality	2.433	1.947	0.033	0.212
Unmarried	2.174	1.327	0.043	0.102
Divorced/Widowed	0.777	0.715	0.030	0.276
Duration of school education (years)				
0–8 years	0.611	1.329	0.012	0.646
**11–12 years**	**2.310**	**1.088**	**0.056**	**0.034**
>12 years	−0.681	1.372	−0.013	0.620
**Smoking status**	**−0.958**	**0.403**	**−0.066**	**0.018**
Insulin (yes)	−0.048	0.732	−0.002	0.948
**Diabetes-related complications**	**−1.335**	**0.327**	**−0.115**	**<0.001**
Comorbidities	−0.621	0.356	−0.046	0.081
**BMI** (kg/m^2^)	**−0.253**	**0.064**	**−0.108**	**<0.001**
Self-reported depression	−0.712	0.869	−0.023	0.413
**MCS at baseline**	**0.106**	**0.029**	**0.105**	**<0.001**
**PCS at baseline**	**0.501**	**0.030**	**0.481**	**<0.001**
HbA1c (%)	−0.103	0.205	−0.014	0.617

Coding of categorical variables is mentioned in the footnote.

*unstandardized regression coefficient; † standard error; ‡ standardized regression coefficient; statistically significant associations are printed in bold

Sex: male and female (referent); Nationality: German nationality (referent), other nationalities. Marital status: “married” (referent), “divorced/widowed” and “unmarried”. Duration of school education: ≤8, 9–10 (referent), 11–12, and > 2 years. Smoking status: variable ranging from 0 to 2, classified into “never”, “former” or “current”. Treatment with insulin: “yes” and “no” (referent). Diabetes-related complications: variable ranging from 0 (no complications) to 4 (four complications). Comorbid diseases: composite variable ranging from 0 to 4. Depression: “no” (referent) and “yes” (depression episode has been diagnosed).

**Table 3** shows the results of the multiple regression analysis for MCS.

The strongest predictors for MCS in diabetes patients after five years were PCS (p = 0.001) and MCS (p<0.001) at baseline. Furthermore, patients who had reported a previous or current depression at baseline had significantly reduced scores in MCS after five years (p = 0.005). No significant association of age, sex, education status, marital status, BMI, and smoking status was detected. There was no temporal association between MCS and glycemic control, nor with treatment status; the number of comorbid diseases was not linked to MCS whereas the number of diabetes-related complications was significantly associated with MCS after five years (p = 0.045).

Additional analyses showed that glycemic control would gain a trend towards statistical significance if the model had not been controlled for diabetes related complications (p = 0.068). Age was not significantly associated with MCS. However, additional subgroup analyses showed that in patients with previous or current depression higher age had a positive impact on MCS – that is, older patients with a previous or current depression episode at baseline had significantly higher MCS at five years later than younger patients with a depression history. In contrast, in the subgroup without previous or current depression at baseline, older patients showed a lower MCS than younger patients.

**Table 4** shows the results of the multiple regression analysis for PCS.

The strongest predictors for PCS at the five-year follow-up were the number of diabetes related complications, PCS, MCS, and BMI at baseline (p<0.001). In addition, smoking (p = 0.018) and age (p<0.001) were significantly predictive for a lower PCS at five years later. A duration of school education of 11–12 years was linked to higher PCS. There was no significant association between PCS and comorbidities including depression, glycemic control, sex, and marital status. Treatment with insulin had no impact on PCS and MCS.

## Discussion

Our findings in a large prospective cohort of elderly patients with diabetes showed a statistically significant relationship of several variables on HRQOL. We found that lower MCS (at baseline), lower PCS (at baseline), a history of depression at baseline and a higher number of complications significantly predict lower MCS at second follow-up. In addition, subgroup analyses showed an interaction effect between depression and age in regard to MCS. A more advanced age predicted higher MCS in people with a reported history of depression. Regarding the prospective association to PCS we found that lower MCS, PCS, higher BMI, smoking, more advanced age and a higher number of complications predict lower PCS at follow-up after five years. No significant association between insulin treatment or glycemic control at baseline in regard to both MCS and PCS at follow-up was found. The results of our longitudinal study expand previous evidence of cross-sectional population-based and clinical studies that investigated the association of several variables with HRQOL in diabetes. Knowledge of temporal predictors of MCS and PCS in diabetes patients is crucial for improving diagnostic and treatment procedures.

### Predictors of mental health

MCS and PCS at baseline were both significantly associated with MCS at follow-up. This reflects the evidence gained from other studies that, in general, HRQOL measured at a specific point in time is a good predictor for HRQOL at a subsequent time measurement point [20]. In addition, we found that self-reported history of depression at baseline was significantly predictive for reduced mental health after five years. Most of the previous studies report cross-sectional findings with negative associations between depression and HRQOL [21]. Prospective data indicate that depression has a negative impact on therapeutic progression [22]. The prospective association between previous depression episodes and decreased MCS at five years later suggests that screening for depression and subsequent treatment may be important issues. Antidepressants are often recommended as the most appropriate treatment in elderly patients with depression. However, a recent meta-analysis showed that the treatment effect of antidepressants in the elderly is rather small [23]. Furthermore, in adults 50 years or older with chronic conditions such as diabetes antidepressants increase the risk of falling [24]. Therefore, beyond development of optimal medication, treatment strategies should take psychosocial aspects into account [25], [26].

To date, the discussion about association between glycemic control and HRQOL in diabetes patients has been controversial. In an RCT study of 275 diabetes patients, Weinberger et al. [12] reported no association between glycemic control and HRQOL at the one year follow-up. A recent cross-sectional study of more than 1000 diabetes patients found a clear association between higher HbA1c levels and decreased HRQOL [8]. In our study, no influence of higher HbA1c levels on HRQOL after five years was noticeable.

### Predictors of perceived physical health

In our study, the presence of diabetes related complications is strongly predictive for decreased HRQOL in diabetes patients after five years. In previous cross-sectional research, this inverse association was consistently reported [9]. We found, in addition, that a higher BMI at baseline and former or current smoking is associated with a lower PCS at five years later - a result that is in line with previous study results. Higher BMI is known to have a negative contemporaneous association to HRQOL in diabetes patients. This relationship appears to be independent of disease severity [27]. In a large multicenter RCT, it was shown that even a weight-management program could improve HRQOL in diabetes patients [28]. In addition to HRQOL, additional complications, and BMI at baseline, higher age was significantly related to lower PCS at five years later. As the PCS sum score is strongly related to the patient's physical ability to perform activities of daily life, this result was to be expected. Interestingly however, age was not associated with MCS at five years later. These results correspond to the findings of previous studies regarding the natural progression in HRQOL which showed that, in older age groups, PCS tends to decline over time whereas MCS still may improve, dependent on gender and age group [29].

### Depression and age-related effects

Additional subgroup analyses showed that in people with a reported history of depression advanced age was significantly associated with higher MCS scores at five years later. What could be the cause of this finding? It has been hypothesized that responsiveness to negative emotions decreases with age, and that older people may, in fact, achieve greater emotional control due to their learning of more effective coping strategies over their lifespan [30], [31]. The reason for this reciprocal interaction between age and depression regarding MCS could be that people have better coping strategies of emotional distress with increasing age.

### Treatment with insulin

Research findings on the association between treatment regimen and HRQOL in people with diabetes are controversial. The United Kingdom Prospective Diabetes Study Group (UKPDS) showed no detectable differences in HRQOL between the different treatment regimens. In contrast, in a cross sectional study that included 2056 individuals from a national sample in the United States, patients on insulin reported lower QOL scores than did those on oral medication [32]. In our study, however, we found no prospective association between insulin treatment and HRQOL. One reason for the controversial findings could be that the relationship between insulin treatment and HRQOL in diabetes patients is heterogeneous across age. In elderly people, insulin treatment may be less stigmatized than in younger patients. The latest research findings show that the omission of insulin injections is negatively correlated with age [33]. It may be possible that the elderly get used to taking insulin and that initial negative appraisal is modifiable over time.

### Strengths and limitations

The major strength of our investigation is a longitudinal design that includes an observation time of five years. In addition, we could maintain the very high response rate of the ESTHER follow-up by using a sophisticated published algorithm for replacing missing values in the SF-12 questionnaire. Furthermore, the analysis of the prospective relationship between baseline variables and HRQOL was controlled for both demographic and lifestyle variables.

However, the study also has several limitations. At baseline we did not assess some highly relevant diabetes-related complications such as retinopathy, autonomic neuropathy and diabetic foot - which could lead to the underestimation of the complication rate as a predicting variable. Nevertheless, the results of our analysis show a strong prospective association between complications and PCS, and respectively, MCS. Another limitation of the study is that we are not able to detect relevant differences that are due to the manifestation time of diabetes.

### Clinical implications

A recently published work of Landman et al. [7] showed that MCS and PCS are inversely associated with mortality in diabetes after a follow-up time of almost ten years. The authors recommend the measurement of HRQOL in clinical practice. Results of our study indicate that interventions intended to improve HRQOL in elderly diabetes patients should address glycemic control, smoking cessation, and weight management, to prevent short term as well as long term complications. In addition, screening of depression appears to be advisable, and treatment of depression should be evaluated in regard to the improvement of MCS in diabetes-patients.
